# Seasonal Dynamics of Mobile Carbon Supply in *Quercus aquifolioides* at the Upper Elevational Limit

**DOI:** 10.1371/journal.pone.0034213

**Published:** 2012-03-30

**Authors:** Wan-Ze Zhu, Min Cao, San-Gen Wang, Wen-Fan Xiao, Mai-He Li

**Affiliations:** 1 Alpine Ecosystem Observation and Experiment Station of Gongga Mountain, Institute of Mountain Hazard and Environment, Chinese Academy of Sciences, Chengdu, China; 2 Tree Physiology Group, Swiss Federal Research Institute WSL, Birmensdorf, Switzerland; 3 State Key Laboratory of Forest and Soil Ecology, Institute of Applied Ecology, Chinese Academy of Sciences, Shenyang, China; 4 Key Laboratory of Tropical Forest Ecology, Xishuangbanna Tropical Botanical Garden, Chinese Academy of Sciences, Mengla, China; 5 College of Agronomy and Biotechnology, Southwest University, Chongqing, China; 6 Key Laboratory of Forest Ecology and Environment, State Forestry Administration, Chinese Academy of Forestry, Beijing, China; Ohio State University, United States of America

## Abstract

Many studies have tried to explain the physiological mechanisms of the alpine treeline phenomenon, but the debate on the alpine treeline formation remains controversial due to opposite results from different studies. The present study explored the carbon-physiology of an alpine shrub species (*Quercus aquifolioides*) grown at its upper elevational limit compared to lower elevations, to test whether the elevational limit of alpine shrubs (<3 m in height) are determined by carbon limitation or growth limitation. We studied the seasonal variations in non-structural carbohydrate (NSC) and its pool size in *Q. aquifolioides* grown at 3000 m, 3500 m, and at its elevational limit of 3950 m above sea level (a.s.l.) on Zheduo Mt., SW China. The tissue NSC concentrations along the elevational gradient varied significantly with season, reflecting the season-dependent carbon balance. The NSC levels in tissues were lowest at the beginning of the growing season, indicating that plants used the winter reserve storage for re-growth in the early spring. During the growing season, plants grown at the elevational limit did not show lower NSC concentrations compared to plants at lower elevations, but during the winter season, storage tissues, especially roots, had significantly lower NSC concentrations in plants at the elevational limit compared to lower elevations. The present results suggest the significance of winter reserve in storage tissues, which may determine the winter survival and early-spring re-growth of *Q. aquifolioides* shrubs at high elevation, leading to the formation of the uppermost distribution limit. This result is consistent with a recent hypothesis for the alpine treeline formation.

## Introduction

Scientists have still not convincingly fully explained the physiological mechanism driving the alpine treeline formation on a global scale. Two dominant hypotheses, the carbon limitation, known also as source limitation, and the growth limitation (also as sink limitation), have received increasing attention in the debate about the alpine treeline phenomenon. The carbon limitation proposes that the alpine treeline trees may suffer from insufficient carbon supplies as a result of a shortage of photo-assimilates caused by low temperatures and short growing seasons [1], [2], and the growth limitation hypothesis states that low temperatures and a short growing season limit cell formation and growth in treeline trees, and carbon supplies are in excess of the growth needs of trees growing at high elevations [3]–[6]. Li *et al.* (2008) indicated that the Himalayan treeline trees suffered from a winter carbon shortage [7], supporting the carbon limitation hypothesis [1], [2], [8]. Similarly, Genet *et al.* (2011) found a significant decrease in NSC contents in roots of *Abies georgei* var. *smithii* at the alpine treeline (4330 m) compared with 3480 m on Mt. Sergyemla, the Tibet-Qinghai Plateau [9]. Susiluoto *et al.* (2010) also reported that the growth of Scots pine (*Pinus sylvestris* L.) in a treeline population in eastern Finnish Lapland, suffered from resources limitation under the current climatic conditions [10]. On the other hand, Hoch *et al.* (2002) [11] and Shi *et al.* (2006, 2008) [12], [13] did not find any decrease in NSC in trees at the alpine treeline compared to low elevation during the growing season, suggesting a growth limitation hypothesis for the alpine treeline formation [5], [6], [11], [13]–[15]. However, their work did not cover the status of carbohydrates during the winter season.

Among all reserves, non-structural carbohydrates (NSC) are the most important food reserve compounds in trees [16]–[18], represent a tree's capital for growth after dormancy and act as a buffer for insufficient source activity [4], [14], [19]. At a whole-tree level, levels of NSC concentration and its pool size indicate a tree's actual carbon-balance between C-gain (photosynthesis) and C-loss (respiration) [12], [14], [19]–[22]. In trees, non-structural carbon is stored mainly in the form of starch, fructosans, soluble sugars and fat [23].

Mobile carbon reserves in trees are known to exhibit seasonal variations [11], [24]. Previous studies showed that the temporal pattern of long-term changes in NSC in tree tissues is an important characteristic that reflects the relative balance between source (net canopy assimilation) and sink activity (the use of assimilates for growth and respiration) [25]–[27]. Generally, carbohydrate reserves of temperate deciduous trees decrease during the spring growth flush, reach a minimum level during early summer, and subsequently increase to a maximum level during late summer and early autumn before the onset of dormancy [24], [28]. During the winter, respiration causes a slight decline, but reserves are still high before growth resumes in the spring [20], [29]–[31]. However, in most cases evergreen species displayed less dramatic seasonal fluctuations in carbohydrate reserves than deciduous species did [15].

Annual patterns of carbohydrate storage and mobilization varied with species, genotypes, and tissues [24], [32]. Different plant species living under similar environmental conditions may show different dynamics of carbohydrate storage, which was found to be correlated with their life forms and ecological strategies [33]–[35]. Seasonal carbohydrate reserve variations have been reported for deciduous trees at low-elevations [30], [35] and conifers in the temperate regions [23], [36], [37]. The temporal variations of NSC in shrubs growing at their uppermost distribution limit have rarely been examined, which limits our understanding of the physiological mechanisms for the phenomenon of shrubs' uppermost limit.

Previous studies have focused on trees rather than shrubs, although just like the alpine treeline, the alpine shrubs (<3 m in height) such as *Quercus aquifolioides* Rehd. et Wils are also distributed to a certain elevational limit and formed an abrupt transition in life-form dominance. *Q. aquifolioides*, an endemic sclerophyllous evergreen broadleaved species, is mainly concentrated in the Hengduan Mountains (93°18′–104°43′E, 26°33′–31°55′N), southwestern China [38]. This species occupies a wide range of habitats and altitudes from 2000 to 4500 m a.s.l., showing a good adaptability to cold, dry habitats with strong UV radiation [39], and, therefore, becomes a late-successional and climax species on sunny, south-facing slopes in that area. Hence, the present study aimed to test the hypothesis that *Q. aquifolioides* shrubs at the uppermost distribution, compared to plants at lower elevations, have higher levels of mobile carbohydrates during the growing season but lower concentrations during winter, as expected by a recently theory of winter C-shortage in treeline trees proposed by Li et al.(2008) [7], [40]. To test this hypothesis, we studied the seasonal variations in mobile carbohydrates and their pool size in *Q. aquifolioides* plants grown at 3000 m, 3500 m, and 3950 m a.s.l. (20 m below the uppermost limit of 3970 m a.s.l.) along an elevational gradient on Zheduo Mt., southwestern China.

## Materials and Methods

The present study was carried out within the study area of the Gong-Ga Mountain Ecosystem Research Station, Institute of Mountain Hazards and Environment. Neither the research species nor the area is legally protected. No specific permits were required for the described study.

### Study site

The study was carried out on a southeast-facing slope of Mt. Zeduo (29°50′–30°16′N, 101°41′–102°6′E, 4298 m a.s.l.) located on the eastern edge of the Qinghai-Tibet Plateau, southwestern China. The climate belongs to the Tibetan Plateau Climate Zone characterized by abundant sunshine, cold winters, and cool summers [41]. The climate data for the sites at 3500 m and 3950 m a.s.l. were calculated using temperature lapse rates derived from climate data measured at the sites of 3000 m a.s.l. (data collected from 2005 to 2009) and in the Kangding meteorological station (2616 m a.s.l., data collected from 1975 to 2009) located directly on the lower slope of the study sites (Table 1). About 75% of the annual precipitation of ∼800 mm occurs between May and September. The soil developed from granite and sandstone is mountain brown soil with a pH value (H_2_O) of 6.54±0.50 and similar fertility on the study slope [42]. The total soil N, P, and K are 1.31±0.11, 0.10±0.07, and 23.48±2.03 mg g^−1^ soil, and the total available soil N, P, and K are 0.13±0.01, 0.01±0.008, and 0.13±0.04 mg g^−1^ soil, respectively (Zhu WZ et al., unpublished data). The soil organic matter is 30.51±2.20 mg g^−1^ soil. There are no statistically significant differences in soil nutrient elements among the sites at the 3 elevations (Zhu WZ et al., unpublished data).

**Table 1:** Characteristics of the sites and the *Quercus aquifolioides* shrub stands studied on a SE-facing slope of Mt. Zeduo, Sichuan, SW China.

		Mean air temperature (°C)*					Shrubs			
Elevation (m a.s.l.)	Slope exposure	Annual	July	January	Annual precipitation (mm)	Length of growing season (days)**	Age (years)	Mean height (m)	Density (clumps ha^−1^)	Canopy coverage
3950	SE	−1.3	5.0	−13.9	No data	157	30–35	1.21±0.11	3300±260	0.75±0.06
3500	SE	1.6	8.7	−10.1	No data	166	30–35	2.08±0.18	3520±310	0.80±0.05
3000	SE	4.7	12.7	−5.8	827.2	179	30–35	2.65±0.25	3650±330	0.85±0.06
2616	SE	7.1	15.8	−2.5	804.5	191	Meteorological station			

* Mean air temperatures at 3500 and 3950 m a.s.l. were calculated using temperature lapse rates derived from data measured at 3000 m (data collected from 2005 to 2009) and 2616 m a.s.l. (data for 1975–2009), for annual, July, and January temperature, respectively.

** Mean growing season length (days with a mean temperature of ≥5°C) at 3500 and 3950 m a.s.l. was derived from measured data at 2616 and 3000 m a.s.l.

*Q. aquifolioides* (oak) stand with an age of 30–35 years old on the sunny slope of Mt. Zeduo is naturally generated since the 1970s, and formed a pure shrub-stand consisting of multi-stem oak clumps (5–10 stems per clump) ranging from 2850 to 3970 m a.s.l. (the uppermost limit). A few individuals of *Picea balfouriana*, *Lyonia ovalifolia*, *Betula platyphylla*, *Rosa moyesii*, and *Sabina procumbens* coexisted in the stand. The understory layer is poorly developed due to the hot and dry conditions. Within the oak shrub stand, we established a 200 meters-long and 20 meters-wide sample strip at each elevation of 3000 m, 3500 m, and 3950 m a.s.l. (20 meters below the uppermost distribution) in April 2008. Characteristics of the study sites/stands are summarized in Table 1.

### Sampling

*Quercus aquifolioides* tissues (leaves, stem, branches, roots) were sampled 7 (current-year tissues) and 11 times (≥1-yr-old tissues) at approximately monthly intervals from April 2008 through October 2009, respectively. The pheonological stage of the oak plants along the elevational gradient was taken into account to determine the sampling time: i.e. (1) April 15–20, 2008/2009 (before bud burst); (2) May 5–10, 2008/2009 (after bud burst); (3) mid-June, mid-July, and mid-August, 2008/2009 (during the growing season); (4) September 15–17, 2008/2009 (at the end of the growing season); (5) October 11–16, 2008/2009, and January 14–19, 2009 (during winter dormancy). If a sampling date was a clear day, samples were then taken around noon to minimize the diurnal NSC fluctuations between samples [7], [43].

For each sampling date, six healthy and undamaged oak clumps (>30 m apart from each other) within each study strip were randomly selected. Roots, stem, branches and leaves were sampled. Roots samples were collected by carefully excavating roots originating from the selected individuals. Root samples were divided into four categories: fine roots (diameter <2.5 mm), medium roots (diameter = 2.5–5 mm), coarse roots (diameter >5 mm), and taproots. Roots samples were washed to remove other materials such as soil particles and dead roots. Each stem sample was a mixture of 3 stem discs taken from the lower, middle and upper one-third stem section of each sample clump. Current-year and previous-year leaves, current-year, 1-year-old, and previous-year (>2 years old) branches were collected from the downslope crown side of each selected clump. Samples for branches, stem and roots included bark, and were cut in small pieces. All samples were immediately stored in a cool box and killed in a microwave oven (40 s at 600 W) and dried to constant mass at 65°C.

### Biomass measurement

In September 2009, six average clumps similar to those used for carbohydrate analyses within each study strip were selected for estimating the biomass of individual clump. Aboveground biomass cut from the ground surface level was separated into different categories corresponding to the tissue category for NSC analysis. Root biomass was investigated by digging out the entire root system, which was also separated into fine roots, medium roots, coarse roots, and taproots, corresponding to those used for NSC analysis. Fresh biomass for each component was measured and recorded. At the individual clump level, dry mass of each biomass component was calculated using the fresh biomass and a content of *dry mass* in an unit of fresh *biomass oven-dried* at 70°C to a constant weight. At the community level, dry biomass of each biomass component was calculated by multiplying mean clump biomass by clump density which was determined in six plots of 20 m×20 m at each elevation.

### Non-structural carbohydrate analysis

Samples were analyzed using the anthrone method to measure starch and soluble sugars [44]. Dried plant material was ground to pass through a 1-mm sieve. The powdered material (0.1 g) was put into a 10-ml centrifuge tube, where 5 ml of 80% ethanol was added. The mixture was incubated at 80°C in a shaking water bath for 30 min, and then centrifuged at 5000 rpm for 5 min. The pellets were re-extracted twice with 80% ethanol. Supernatants were retained, combined and stored at −20°C for soluble sugar determinations. Extracted soluble sugars were determined spectrophotometrically using the anthrone method at 625 nm within 30 min [44]. Soluble carbohydrate concentrations were calculated from standard regression equations based on glucose standard solutions. The soluble sugar concentration was calculated on a dry matter basis (% d.m.).

Starch was extracted from the ethanol-insoluble pellet after removing the ethanol by evaporation. Starch in the residue was released by boiling in 2 ml distilled water for 15 min in a boiling water bath. After cooling to room temperature, 2 ml 9.2 M HClO_4_ was added and the starch was hydrolyzed for 15 min. 4 ml distilled water was added and the mixture centrifuged at 5000 g for 10 min. The pellets were extracted one more time with 2 ml 4.6 M HClO_4_. The combined supernatants were adjusted to 20 ml. Starch concentration was measured spectrophotometrically at 625 nm using anthrone reagent, and calculated by multiplying the glucose concentration by the conversion factor of 0.9 [45]. Glucose was used as a standard. The starch concentration was expressed on a dry matter basis (% d.m).

### Data analysis

Nonstructural carbohydrate is defined as the sum of the starch plus the total soluble sugars within each tissue sample for each sample date. The NSC pool size was calculated by multiplying tissue biomass by their corresponding mean NSC concentrations in each sample.

All statistical tests treated individual shrub clump as replicates (*n* = 6). Normality of distribution and homogeneity of data (NSC, soluble sugars, starch) were checked (Kolmogorov-Smirnov-Test) before any further statistical analysis. Two-way repeated ANOVAs were performed with elevation and sampling date as factors to determine the effects of elevation and sampling date on concentrations of NSC and its components for each tissue type. Differences in mean concentrations of NSC and its components, biomass, and NSC-pools for each tissue type were compared by using the least significant ranges test at 5% level. The SPSS (version 17.0) statistical software (SPSS Inc. Chicago, USA) was used to perform the analyses.

## Results

### Seasonal dynamics of tissue non-structural carbohydrates

**Figure 1:**
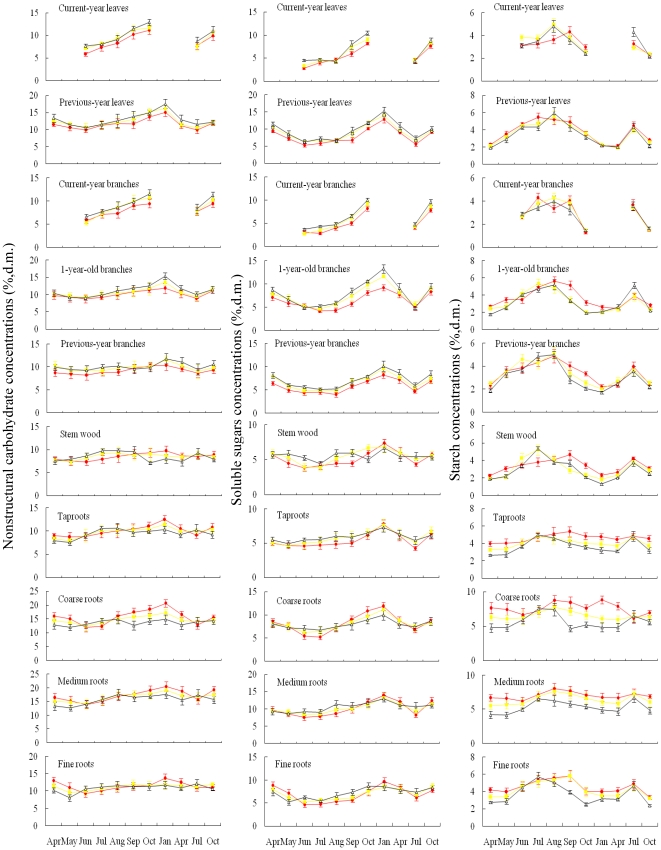
Mean concentrations (±1SE, *n* = 6, % d.m.) of nonstructural carbohydrates (left panels), soluble sugars (middle panels), and starch (right panels) in relation to sampling time for tissues of *Quercus aquifoliodes* shrubs grown at 3000 m, 3500 m, and 3950 m a.s.l. on the Mt. Zheduo, SW China. Symbols: • (red) = 3000 m a.s.l.; ▪ (yellow) = 3500 m a.s.l.; Δ (black) = 3950 m a.s.l.

With an exception of NSC in stem wood, concentrations of soluble sugars, starch, and NSC in each tissue varied significantly with sampling dates (*P*<0.001 for sugars in all tissues; *P*<0.05 for starch and NSC in all tissues) (Table 2, Fig. 1). Only in two cases significant interactions of elevation×sampling date were found, i.e. for NSC in coarse roots (*P* = 0.011) and for soluble sugars in 1-year-old branches (*P* = 0.033) (Table 2). In all other cases, there were no elevations×sampling date interactions on concentrations of NSC, sugars, and starch in tissues (Table 2), indicating that the patterns of seasonal variations in NSC and its components within each tissue type were similar in oak plants grown at different elevations (Fig. 1).

**Table 2:** Analysis of variance of the effects of elevations (3000 m, 3500 m and 3950 m a.s.l.) and sampling time (from April 2008 to October 2009) on concentrations of nonstructural carbohydrate (NSC) and its components (sugars, starch) in different tissues of *Quercus aquifolioides* shrubs.

			Soluble sugars		Starch		NSC	
Tissues	Factors	d.f.	*F*	*P*	*F*	*P*	*F*	*P*
Current-year leaves	Elevations (E)	2	9.280	**<0.001**	0.103	0.902	2.930	0.061
	Sampling time (ST)	6	73.075	**<0.001**	4.107	**0.002**	13.401	**<0.001**
	E×ST	12	1.902	0.051	0.617	0.820	0.149	1.000
Previous-year leaves	Elevations (E)	2	15.811	**<0.001**	0.519	0.597	3.343	**0.040**
	Sampling time (ST)	10	43.474	**<0.001**	9.377	**<0.001**	7.356	**<0.001**
	E×ST	20	0.645	0.868	0.218	1.000	0.151	1.000
Current-year branches	Elevations (E)	2	11.442	**<0.001**	0.175	0.839	3.603	**0.033**
	Sampling time (ST)	6	76.779	**<0.001**	13.697	**<0.001**	13.349	**<0.001**
	E×ST	12	0.549	0.873	0.515	0.897	0.293	0.988
1-year-old branches	Elevations (E)	2	22.597	**<0.001**	3.447	**0.036**	6.746	**0.002**
	Sampling time (ST)	10	57.951	**<0.001**	23.758	**<0.001**	13.132	**<0.001**
	E×ST	20	1.802	**0.033**	1.455	0.120	0.626	0.882
Previous-year branches	Elevations (E)	2	20.093	**<0.001**	1.568	0.215	5.567	**0.005**
	Sampling time (ST)	10	31.911	**<0.001**	14.947	**<0.001**	2.812	**0.005**
	E×ST	20	0.342	0.996	0.568	0.924	0.314	0.998
Stem wood	Elevations (E)	2	1.518	0.225	3.085	**0.05**	0.026	0.974
	Sampling time (ST)	10	4.505	**<0.001**	13.269	**<0.001**	1.285	0.251
	E×ST	20	0.877	0.615	1.577	0.075	0.736	0.780
Taproots	Elevations (E)	2	1.747	0.180	6.634	**0.002**	0.609	0.546
	Sampling time (ST)	10	4.897	**<0.001**	2.316	**0.018**	2.814	**0.004**
	E×ST	20	0.216	1.000	0.293	0.999	0.302	0.998
Coarse roots	Elevations (E)	2	0.255	0.775	24.149	**<0.001**	14.645	**<0.001**
	Sampling time (ST)	10	14.036	**<0.001**	2.220	**0.022**	8.041	**<0.001**
	E×ST	20	0.872	0.621	1.408	0.137	2.054	**0.011**
Medium roots	Elevations (E)	2	0.739	0.480	20.21	**<0.001**	3.941	**0.023**
	Sampling time (ST)	10	10.103	**<0.001**	3.68	**<0.001**	7.951	**<0.001**
	E×ST	20	0.619	0.890	0.271	0.999	0.765	0.748
Fine roots	Elevations (E)	2	0.787	0.458	3.382	**0.038**	1.065	0.349
	Sampling time (ST)	10	16.984	**<0.001**	4.856	**<0.001**	2.678	**0.006**
	E×ST	20	1.454	0.115	0.289	0.999	0.895	0.594

Statistical significances were tested for each tissue category using two-way ANOVAs with elevation and sampling time as factors. Significant differences (*P*<0.05) are highlighted in bold.

**Figure 2:**
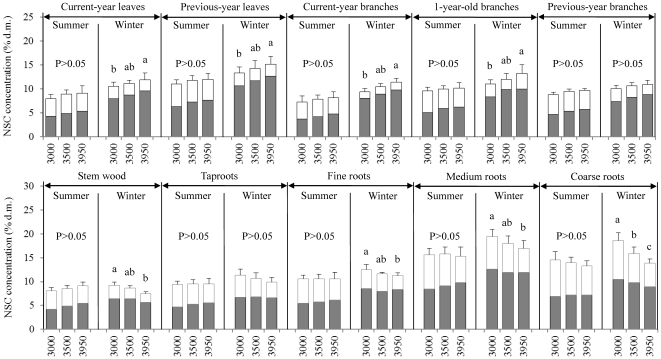
Mean concentrations of soluble sugars (dark gray), starch (white), and non-structural carbohydrates (NSC = soluble sugars+starch) in tissues of *Quercus aquifolioides* grown at 3000 m, 3500 m, and 3950 m a.s.l. during the growing season (May–September) and the dormant season (October–April), calculated using data measured across an one-year cycle from May 2008 to April 2009. Statistical differences in means among elevations within each tissue category and season were tested by t-paired comparison. Different letters indicate statistically significant differences (p<0.05) in NSC among elevations. Standard error bars (+1SE) are given for NSC only.

Within each tissue type and elevation where plants grown, concentrations of NSC and soluble sugars in tissues tended to be higher during the dormant season than those during the growing season (Figs. 1 and 2), but tissue starch concentrations showed lower levels during the dormant season than during the growing season (Figs. 1 and 2). Concentrations of sugars peaked during winter (January), and remained at a higher level up to bud flush, and then decreased to reach a minimum level in summer (June–August), whereas starch concentrations increased after winter dormancy, and peaked during the mid-growing season (July–August), then gradually declined to reach a minimum level in deep winter and early spring (Fig. 1). Pooled data across tissue types and elevations showed, for example, that the mean concentrations of NSC and sugars were 10.63% and 5.98% during the growing season (May–September), and 12.15% and 8.69% during the winter dormancy (October–April), respectively (*P*<0.001 for both NSC and sugars), whereas the mean starch content was 4.65% during the growing season and 3.46% during the dormant season (*P*<0.001) (data not shown).

### Changes in non-structural carbohydrates along the elevational gradient

**Figure 3:**
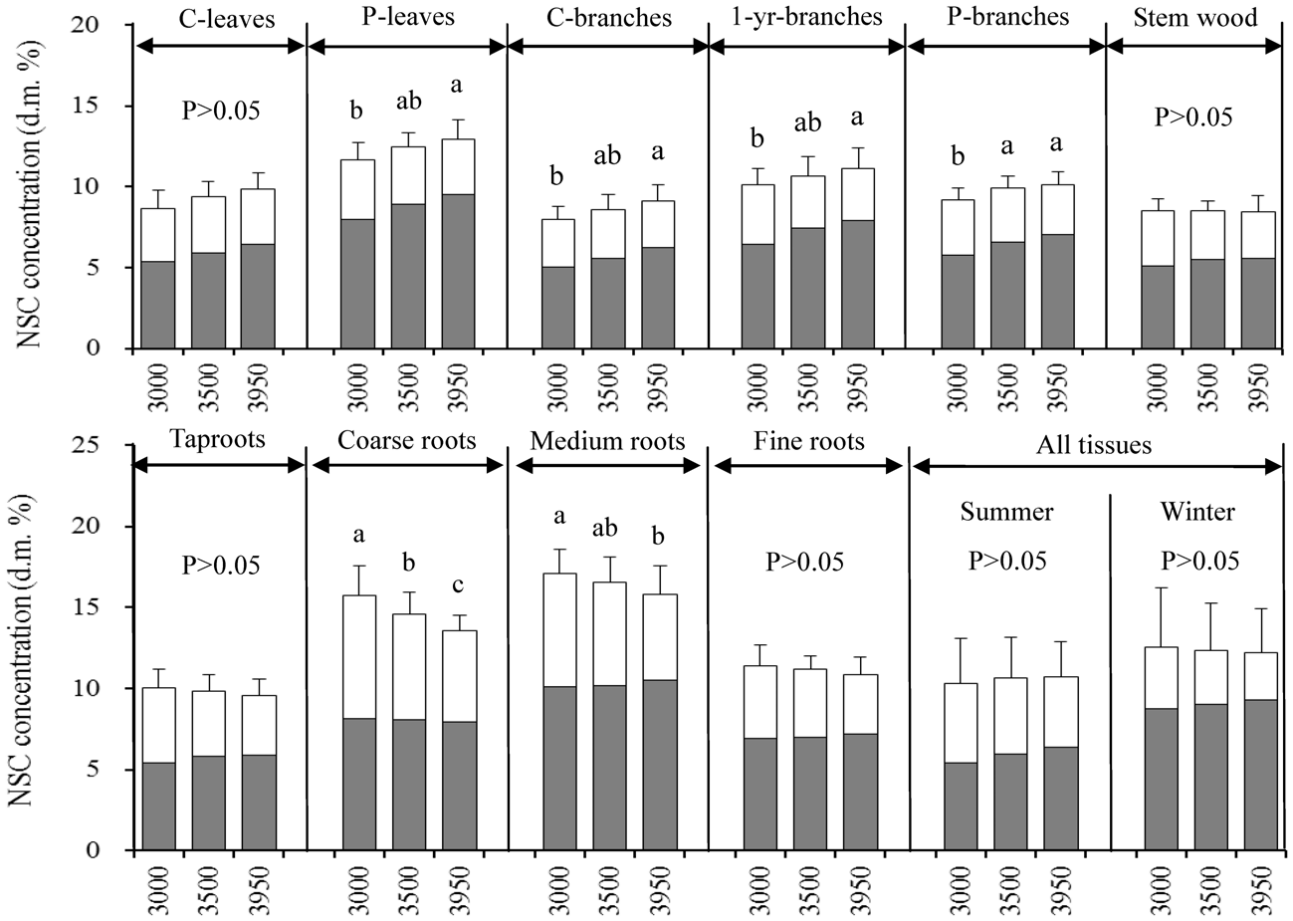
Mean concentrations (calculated using pooled data across sampling dates; *n* = 6) of soluble sugars (dark gray), starch (white), and non-structural carbohydrates (NSC = soluble sugars+starch) in tissues of *Quercus aquifolioides* plants grown at 3000, 3500, and 3950 m a.s.l. on a SE-facing slope of Mt. Zheduo, Sichuan, SW China. Different letters indicate statistically significant differences (*p*<0.05) in NSC concentration among elevations. Standard error bars (+1SE) are given for NSC only.

The concentrations of soluble sugars significantly increased with increasing elevation in current-year leaves (*P*<0.001), previous-year leaves (*P*<0.001), current-year branches (*P*<0.001), 1-year-old branches (*P*<0.001), and previous-year branches (*P*<0.001) (Table 2, Fig. 3). Also, elevation significantly influenced the concentrations of starch in 1-year-old branches (*P* = 0.036), stem (*P* = 0.05), taproots (*P* = 0.002), coarse roots (*P*<0.001), medium roots (*P*<0.001), and fine roots (*P* = 0.038) (Table 2). The tissue NSC concentrations were found to be significantly affected by elevation for previous-year leaves (*P* = 0.04), current-year branches (*P* = 0.033), 1-year-old branches (*P* = 0.002), previous-year branches (*P* = 0.005), coarse roots (*P*<0.001), and medium roots (*P* = 0.023) (Table 2).

The elevational effects on starch and NSC concentrations were more marked during the winter season (October–April) rather than during the growing season (May–September) (Fig. 2). During the growing season, NSC concentrations in plant tissues did not change with increasing elevation. However, during the winter season, NSC concentrations significantly increased in leaves and younger branches, but significantly decreased in stem and roots with increasing elevation (Fig. 2). Similarly, starch concentrations in plant tissues (except for the medium roots) did not vary with elevations during the growing season, but significantly decreased (except for leaves and current branches) with increasing elevation during the dormant season (Fig. 2). Concentrations of soluble sugars in stem wood and roots tended to decrease with increasing elevation during the winter season, but to increase in other tissues during both seasons (Fig. 2).

The pooled data across sampling dates indicated that tissue NSC concentrations tended to increase with increasing elevation in leaves and branches, but to decrease in stem wood and roots (Fig. 3). Starch contents in leaves and current year branches did not vary with elevation, but significantly decreased with increasing elevation in stem and roots, whereas concentrations of soluble sugars in all tissues tended to increase with increasing elevation (Fig. 3).

The ratio of soluble sugars to starch within each tissue showed similar patterns of increased trend with increasing elevation for both seasons (except for the current year leaves at 3500 m during the growing season) (Table 3). Plants grown at higher elevations or in winter had higher ratios of sugars to starch contents compared to lower elevations or summer, respectively (Table 3). Within each elevation and each tissue type, the ratio was higher in winter than that in summer (Table 3). Across elevations, the ratio lay between 1.0 and 1.5 in all plant tissues during the growing season, but between 3 and 6 in leaves and branches, and between 1.5 and 3 in stem wood and roots during the winter season (Table 3). Within the growing season, the average increment of the sugar-starch ratios in plants reached to 17.1% (ranging from 7 to 32%, except for the current year leaves) at 3500 m, and 36.0% at 3950 m compared to at 3000 m a.s.l., respectively (Table 3). Within the winter season, that increment percent reached to 18.9% at 3500 m and 37.5% at 3950 m compared to at 3000 m a.s.l. (Table 3). Those tissue-, elevation-, and time-dependent sugar-starch ratios indicated that the relative contributions of sugars and/or starch to NSC varied among tissue types, elevations, and with time (Table 3). The mean contributions of sugars to NSC increased with increasing elevation for both growing season (53%, 57%, and 61% for 3000, 3500, and 3950 m, respectively) and dormant season (74%, 77%, and 79% for 3000, 3500, and 3950 m, respectively), and those relative contributions were higher in winter than in summer within each elevation (Table 3).

**Table 3:** Concentration ratios of total soluble sugars to starch (RSS) in tissues of *Quercus aquifolioides* shrubs grown at elevations of 3000 m, 3500 m, and 3950 m a.s.l. (the elevational limit) during the growing season (May–September) and the dormant season (October–April).

Season	Growing season (May–September)					Dormant season (October–April)				
Elevation (m a.s.l.)	3000	3500		3950		3000	3500		3950	
Ratio & increment	Ratio	Ratio	+%	Ratio	+%	Ratio	Ratio	+%	Ratio	+%
Current-year leaves	1.21	1.19	−1.9	1.43	18.2	3.03	3.56	17.3	4.20	38.4
Previous-year leaves	1.33	1.64	23.0	1.75	31.3	4.04	4.55	12.5	5.17	27.9
Current-year branches	1.23	1.61	31.5	2.02	64.4	5.81	5.84	0.6	6.31	8.6
One-yr-old branches	1.12	1.44	28.3	1.57	39.5	3.09	4.66	50.9	5.02	62.7
Previous-year branches	1.14	1.30	13.8	1.45	27.2	2.81	3.43	22.0	4.24	50.8
Stem wood	1.12	1.31	17.3	1.50	34.0	2.29	2.98	30.0	3.20	39.6
Taproots	1.01	1.26	24.2	1.39	36.9	1.42	1.76	24.0	2.03	42.5
Coarse roots	0.89	1.05	17.7	1.20	34.1	1.29	1.59	23.2	1.80	39.6
Medium roots	1.30	1.39	7.3	1.88	45.1	1.85	1.94	4.6	2.41	30.0
Fine roots	1.09	1.19	9.6	1.40	28.9	2.12	2.20	3.6	2.86	34.4
Mean sugar-starch ratio	1.14	1.34	17.1	1.56	36.0	2.78	3.25	18.9	3.72	37.5
Sugar contribution to NSC (%)	53.3	57.3	7.0	60.9	14.3	73.5	76.5	4.1	78.8	7.2

Note (1) Increment (%) was defined as the increment percent of ratio at higher elevation compared to that at 3000 m a.s.l., and (2) ratios were calculated using data shown in Figure 2.

### Total nonstructural carbohydrate pools at individual and community level

**Figure 4:**
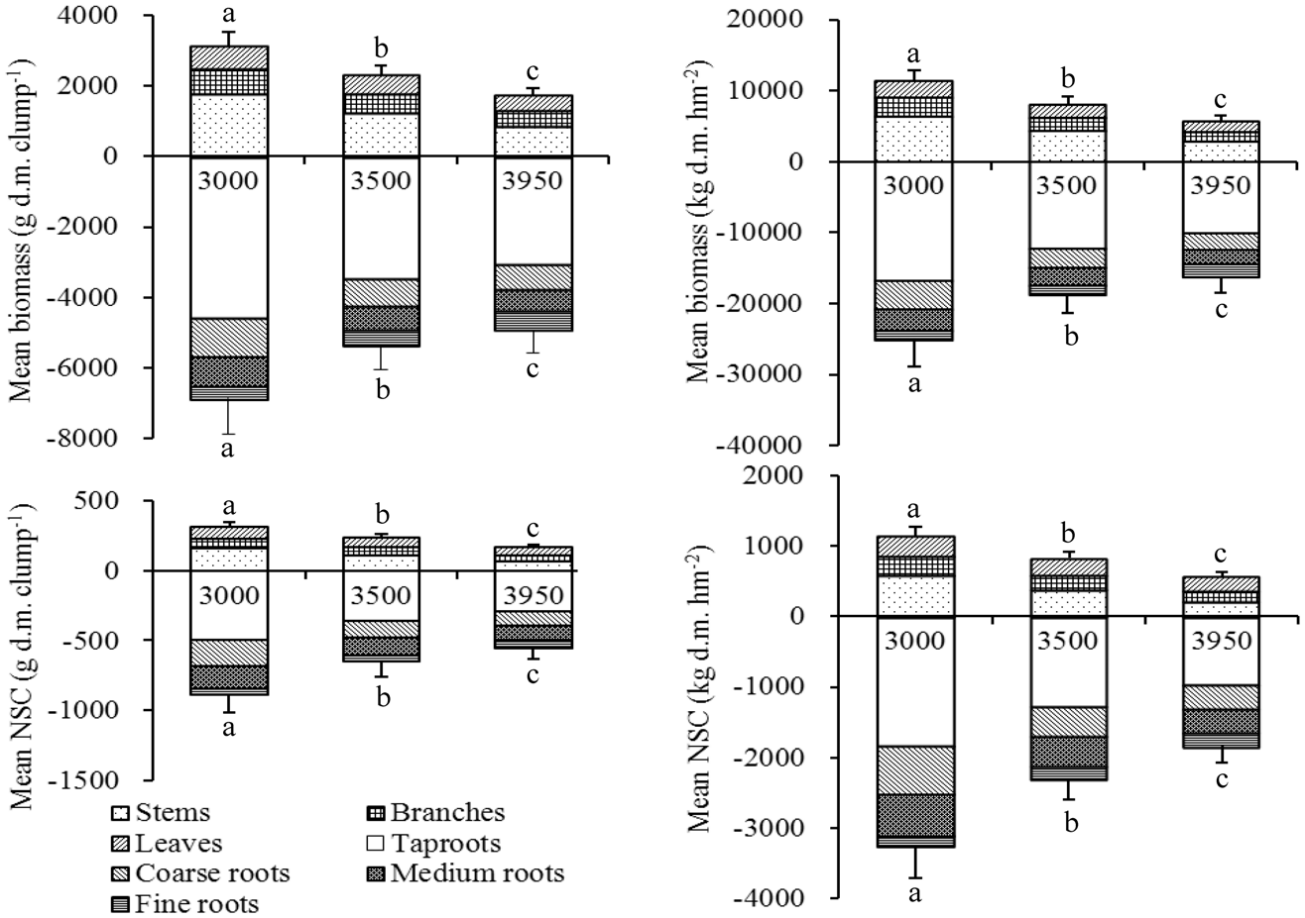
Mean biomass and pool size of non-structural carbohydrates (NSC) at individual and stand level (both *n* = 6) of *Quercus aquifolioides* shrubs grown at 3000, 3500, and 3950 m a.s.l. on a SE-facing slope of Mt. Zheduo, SW China. Different letters indicate statistically significant differences (*p*<0.05) in biomass or NSC pool size among elevations. Standard errors bars (+1SE) are given for the total above- or below-ground biomass or the total above- or below-ground NSC pool size.

The biomass at tissue level showed a decreasing trend with increasing elevation, except the fine roots biomass increased with increasing elevation (Fig. 4). Correspondingly, both the total aboveground biomass and the total belowground biomass at individual clump and community level decreased significantly with increasing elevation (Fig. 4). With increasing elevation, plants invested more fixed carbon to belowground part. The ratio of the belowground biomass to the aboveground biomass increased with increasing elevation from 2.20 at 3000 m, to 2.30 at 3500 m, and 2.81 at 3950 m a.s.l. at both individual clump and community level (Fig. 4).

At the tissue level, the NSC pool size tended to decrease with increasing elevation, except the NSC pool size in fine roots increased significantly with increasing elevation (Fig. 4). Correspondingly, both the total aboveground NSC pool size and the total belowground NSC pool size at individual clump and community level decreased significantly with increasing elevation (Fig. 4). The belowground NSC storage seemed to play more important roles in determining plant life at its upper elevational limit, where the belowground NSC pool size reached >3 times the aboveground NSC pool size (Fig. 4). Across the elevational gradient, the ratios of belowground NSC pool size to aboveground pool size were similar ranging from 2.86 at 3000 m, and 2.78 at 3500 m, to 3.26 at 3950 m a.s.l. (Fig. 4).

## Discussion

### Overall elevational trends in nonstructural carbohydrates

The present study showed that tissue mobile carbohydrate concentrations varied with time (Fig. 1), and elevational effects on NSC occurred in the dormant season rather than in the growing season (Fig. 2), indicating season-dependent effects of elevation on levels of NSC (Tables 2 and 3). Similarly, previous studies suggested that the late-season levels of tissue NSC may be most appropriate to assess environmental effects on carbon allocation affecting winter survival and re-growth of coniferous populations at high elevations [46], [47], and to test the carbon limitation or growth limitation hypothesis for the mechanisms of the alpine treeline formation [4], [48].

Levels of NSC concentrations within each tissue type did not show any differences in plants grown at the upper elevational limit compared to those at lower elevations during the growing season (Fig. 2). Similarly, Hoch and Körner (2003) [4], and Shi *et al.* (2006, 2008) [12], [13] also did not find any disadvantages in mobile carbohydrates in trees grown at the alpine treelines in summer. Li *et al.* (2009) found that two coniferous species (*Abies georgei*, *Juniperus saltuaria*) grown at the timberline (4300–4400 m a.s.l.) had a greater relative abundance of NSC compared to the same species grown at lower elevations in southeastern Tibetan Plateau in the growing season [49]. Those studies indicated no carbon limitation in treeline trees during the photosynthetically active time. But those studies did not cover the NSC status in trees during the dormant season. Li *et al.* (2008) found that the treeline coniferous trees (*Abies fabri, Picea balfouriana*) suffered from a ‘carbon limitation’ during and after dormancy in winter and in the early spring, but not during the growing season [7], [40]. However, they did not distinguish whether aboveground or belowground tissues in the treeline trees suffered from a carbon limitation in winter [7], [40].

Pooled data in the present study found that leaves and branches showed an increasing trend but stem and roots exhibited a decreasing trend of tissue NSC concentrations with increasing elevation (Fig. 3). In consistent with the present study, Shi *et al.* (2006) showed that not leaves and branches but roots of four woody species tended to decrease the mobile carbohydrates concentrations with increasing elevation up to their elevational limit at the end of growing season (see their Fig. 2 on page 374) [12]. Again, Genet *et al.* (2011) found that roots of *Abies georgei* trees grown at the alpine treeline (4330 m a.s.l.) had significantly lower NSC concentration (*P*<0.001) than those at 3480 m a.s.l. on Sergyemla Mt., SW China, during the growing season [9]. Our study found that not during the growing season but in the dormant season, storage tissues (stem and roots) of plants at the upper elevational limit had significantly lower levels of NSC, compared to those at lower elevations (Fig. 2), which may suggest the significance of winter reserve in storage tissues in determining the winter survival and spring re-growth of plants at the upper elevational limit.

Except for the season-dependent elevational effects, the present study also found that the elevational effects on levels of NSC and its components were tissue-dependent (Tables 2, Figs. 2 and 3). Significant elevational effects on soluble sugars were found for leaves and branches but not for stem and roots, whereas elevation significantly affected starch concentrations in stem and roots but not in leaves and branches (Table 2). These results suggested that the ten tissue types analyzed may be divided into two groups: a source group including leaves and branches which are the source of soluble sugars, and a sink group including stem and roots which absorb and use those sugars. Indeed, the bark of younger branches of *Q. aquifoliodes* is green and allows bark photosynthesis to produce glucose. For example, light-saturated photosynthetic rate of current-year braches in *Quercus robur* was found to reach 1.89 µmol m^−2^ s^−1^ in winter and 2.73 µmol m^−2^ s^−1^ in summer [50], indicating that bark photosynthesis can contribute to an important proportion of the whole-plant carbon balance [51]–[53].

Both source and sink tissue influence the carbon balance at a tree level [48]. Previous studies suggested that plants in a harsh environment prefer to store more NSC so as to increase their survival rather than investing it to growth [54]. The observed decline in shrub height with increasing elevation (Table 1) seemed not to be caused by a carbon limitation during the growing season but by a shorter growing period (Table 1) because tissue NSC concentrations did not decrease with increasing elevation during the growing season (Figs. 1 and 2). Previous studies also showed that the ring-porous oak species such as *Q. aquifoliodes* trees need more nonstructural carbohydrate during winter for their higher maintenance respiration and higher basal respiration rate of sapwood [34], [55], [56]. Also, in other species such as *Abies lasiocarpa*, Bansal and Germino (2008) observed greater NSC concentrations in the aboveground tree tissues at the upper limit when respiration decreased [48]. On the other hand, Reader (1978) [57] and Chapin *et al.* (1980) [58] stated that evergreen species retained leaf resources *in situ* rather than translocating them to stem and roots during winter. These may be the reasons for why decreased NSC concentrations with increasing elevation were found in stem and roots of evergreen plants in the present study and elsewhere [9], [13].

The total NSC pool size at individual level was considered to better answer the question of whether the alpine treeline trees physiologically suffer from a carbon limitation, because the treeline trees may compensate for decreases in plant size by accumulating more NSC in plant tissues [19], [59]. The plant size (Table 1) and biomass (Fig. 4) decreased with increasing elevation significantly, and the NSC concentrations in leaves and branches increased but those in stem and roots decreased with increasing elevation (Fig. 3). Consequently, the plant NSC pool size decreased significantly with increasing elevation (Fig. 4). But these decreased NSC concentration and pool size did not imply fully depletion of mobile carbohydrates in plants grown at the elevational limit.

The ratio of sugars to starch may help to explain the alpine treeline phenomenon [7], [40]. Li *et al.* (2008) proposed that trees growing at the elevational or latitudinal climate limit rely not only on the total NSC concentration, but also require a sufficiently high sugar-starch ratio to overwinter successfully [7], [40]. Previous studies indicated that trees' hardiness and ability to withstand cold were positively related to soluble sugars concentration in perennial organs, and that an increase in proportion of soluble sugars reflected an adaptation through adjusting intracellular osmotic concentration [7], [28], [46], [60]. Li *et al.* (2008) suggested that trees at their upper elevational limit need a minimum sugar-starch ratio of about three to overwinter [7]. The sugar-starch ratios gained in the present study supported Li *et al.*'s suggestion [7], showing an increasing trend with increasing elevation and higher values in winter (mean ratio of 2.78, 3.25, and 3.72 in plants at 3000, 3500, and 3950 m a.s.l., respectively) and lower values in summer (mean ratio of 1.14, 1.34, and 1.56 in plants at 3000, 3500, and 3950 m a.s.l., respectively) (Table 3). No other empirical and experimental data can be found in the literature related to sugar-starch ratios in woody plants at their elevational limit. Patton *et al.* (2007) found that the sugar-starch ratio in zoysiagrass (*Zoysia spp*.) was significantly positively correlated with cold hardiness [61]. Strand *et al.* (2003) also found that the sugar-starch ratio in leaves of *Arabidopsis thaliana* grown at 23°C was much lower (<1.5) than in leaves of plants grown at 5°C (up to 2.5) [62].

### Seasonal dynamics of non-structural carbohydrates in plants

In line with previous studies [63]–[66], our data showed that tissue NSC concentrations of *Q. aquifoliodes* plants varied significantly with season (Table 2), and the seasonal changes in NSC concentrations of various tissues followed similar cyclic patterns (Fig. 1). This result may mainly reflect the season-dependent balance between carbon gain (photosynthesis) and carbon loss (growth and maintenance respiration) of *Q. aquifolioides*. Concentrations of soluble sugars in tissues were higher during the dormant season than those during the growing season (Figs. 1 and 2), but tissue starch concentrations were lower during the dormant season than the growing season (Figs. 1 and 2), which may result from the conversion between glucose and starch. Ericsson (1978) pointed out that excess sugars accumulated as non-structural starch when C production exceeds growth demands, and conversely provided a buffer when consumption is greater than current production [67]. *Q. aquifolioides* plants having high photosynthetic rate during the growing season converted excess glucose into starch for storage, leading to higher levels of starch concentration in July and August (Fig. 1). After the peak growing season, plant's photosynthetic rate declined as the environmental temperature decreased, and the stored starch was gradually broken down into glucose, leading to decreased starch but increased sugars concentrations to maintain a higher level of soluble sugars in plant tissues (Fig. 1, Table 3). Krueger and Trappe (1967) also found that sugars concentration in Douglas-fir plants increased starting in November, reached up to three times the lowest (summer) concentration, then decreased gradually to a low level in May, whereas the starch concentration firstly decreased and then increased during the same time in winter [68]. These increased concentrations of soluble sugars prevent intracellular ice formation which can severely injure plants [60], [69]–[71]. Hence, sugar-starch ratios were higher during the dormant season than the growing season (Table 3).

Tissue NSC concentrations declined from winter to the early growing season (Fig. 1), suggesting that the mobile carbohydrate reserves and recently formed photosynthates in previous-year leaves supported budbreak and shoot growth in the early spring. These results supported both Chapin *et al.*'s (1990) hypothesis [16] that old leaves of evergreen species have a primary role in resource remobilization, and Barbaroux and Bréda's hypothesis (2002) that early growth before budburst in *Quercus petraea* trees is dependent on storage compounds [34]. Cherbuy *et al.* (2001) demonstrated that both source tissues and sink tissues of mature evergreen *Quercus ilex* participated in supplying carbon for re-growth in the early spring [72], which may differ from deciduous species [73]. Schädel *et al.* (2009) suggested that evergreen trees relied on mobile carbon reserves to a lesser extent than deciduous trees during budbreak [74]. Kramer and Kozlowski (1979) indicated that evergreen trees accumulated NSC in needles prior to bud-break, and mobilized them during the initiation of shoot growth [24]. Jonasson (1989, 1995) found that old leaves may not be the only place of remobilization in evergreen species [75], [76]. Our data indicated that the reduction of NSC concentration during budbreak in May, compared to April, reached −10.86% in previous-year leaves, −7.30% in branches, −8.12% in belowground roots, and −1.75% in stem, suggesting high NSC remobilization from both source and sink tissues for re-growth. Hence, roots carbohydrate storage may be considered to be essential for persistence and development of *Q. aquifolioides* at its elevational limit. It is, therefore, most possible that significantly lower winter NSC storage in sink tissues at the elevational limit, compared to lower elevations (Figs. 2 and 3), may determine the winter survival and early-spring re-growth of *Q. aquifolioides* shrubs at high elevation.

### Conclusion

Levels of mobile carbohydrates concentrations in *Q. aquifolioides* shrubs grown along the elevational gradient varied significantly with season (Fig. 1, Table 2), which reflected the season-dependent carbon balance between carbon gain (photosynthesis) and carbon loss (growth and maintenance respiration). Tissue NSC concentrations were lowest at the beginning of the growing season (Fig. 1), which indicated that plants used the winter reserve storage for re-growth in the early spring. Storage tissues, in particular roots, had significantly lower winter starch and NSC concentrations in plants grown at their elevational limit compared to lower elevations (Figs. 1 and 2). The present study suggests the significance of winter reserve in storage tissues, which may determine the winter survival and early-spring re-growth of *Q. aquifolioides* shrubs at high elevation, leading to the formation of the uppermost distribution limit. This result is consistent with a recent hypothesis that not summer but winter carbon shortage determines tree growth at high elevation (*Abies fabri* and *Picea balfouriana* var. *hirtella*), resulting in the formation of the alpine treeline [7], [40].
